# Inactivation of GIRK channels weakens the pre‐ and postsynaptic inhibitory activity in dorsal raphe neurons

**DOI:** 10.14814/phy2.13141

**Published:** 2017-02-14

**Authors:** Nerea Llamosas, Luisa Ugedo, Maria Torrecilla

**Affiliations:** ^1^ Department of PharmacologyFaculty of Medicine and NursingUniversity of the Basque CountryUPV/EHULeioaSpain

**Keywords:** Depression, dorsal raphe, GABA, GIRK, IPSC

## Abstract

The serotonergic tone of the dorsal raphe (DR) is regulated by 5‐HT_1A_ receptors, which negatively control serotonergic activity via the activation of G protein‐coupled inwardly rectifying K^+^ (GIRK) channels. In addition, DR activity is modulated by local GABAergic transmission, which is believed to play a key role in the development of mood‐related disorders. Here, we sought to characterize the role of GIRK2 subunit‐containing channels on the basal electrophysiological properties of DR neurons and to investigate whether the presynaptic and postsynaptic activities of 5‐HT_1A_, GABA_B_, and GABA_A_ receptors are affected by *Girk2* gene deletion. Whole‐cell patch‐clamp recordings in brain slices from GIRK2 knockout mice revealed that the GIRK2 subunit contributes to maintenance of the resting membrane potential and to the membrane input resistance of DR neurons. 5‐HT_1A_ and GABA_B_ receptor‐mediated postsynaptic currents were almost absent in the mutant mice. Spontaneous and evoked GABA_A_ receptor‐mediated transmissions were markedly reduced in GIRK2 KO mice, as the frequency and amplitude of spontaneous IPSCs were reduced, the paired‐pulse ratio was increased and GABA‐induced whole‐cell currents were decreased. Similarly, the pharmacological blockade of GIRK channels with tertiapin‐Q prevented the 5‐HT_1A_ and GABA_B_ receptor‐mediated postsynaptic currents and increased the paired‐pulse ratio. Finally, deletion of the *Girk2* gene also limited the presynaptic inhibition of GABA release exerted by 5‐HT_1A_ and GABA_B_ receptors. These results indicate that the properties and inhibitory activity of DR neurons are highly regulated by GIRK2 subunit‐containing channels, introducing GIRK channels as potential candidates for studying the pathophysiology and treatment of affective disorders.

## Introduction

The dorsal raphe (DR) is a serotonergic (5‐HT) midbrain nucleus that is highly implicated in the etiology of several mood disorders, including depression (Jans et al. 2007; Olivier 2015). The activity of this nucleus is critically regulated by 5‐HT_1A_ receptors, which, upon stimulation, activate G protein‐coupled inwardly rectifying K^+^ (GIRK) channels, leading to a reduction in membrane excitability and an inhibition of action potentials (Aghajanian and Lakoski 1984; Bayliss et al. 1997). In addition to 5‐HT_1A_ receptor‐mediated regulation, the GABAergic system also provides inhibitory control of local DR activity and descending excitatory inputs (Gervasoni et al. 2000; Celada et al. 2001; Varga et al. 2001, 2003; Amat et al. 2005; Challis et al. 2014).

Recently, emerging evidence has shown that GABA_A_ and GABA_B_ receptor‐mediated neurotransmissions of the DR are highly involved in drug‐related stress, aggression, social avoidance, abnormal responses to social stimulation, and stress‐related psychiatric disorders, such as depression (Takahashi et al. 2010, 2012; Challis et al. 2013; Araki et al. 2016; Li and Kirby 2016). In addition, the manipulation of 5‐HT_1A_ autoreceptors in the DR affects 5‐HT neuronal firing, determines the responsiveness to stress in depression‐related tasks and alters behavioral responses to antidepressant drugs in mice (Richardson‐Jones et al. 2010). Interestingly, the recent phenotypic and electrophysiological characterization of GIRK2 knockout mice revealed that the mutation promotes depression‐resistant behavior, reduced antidepressant responses, increased 5‐HT neuronal firing, and decreased 5‐HT_1A_ receptor activity in the DR in vivo (Llamosas et al. 2015). Although it was reported that GIRK channels located in the DR are coupled to 5‐HT_1A_ and GABA_B_ receptors (Innis et al. 1988; Williams et al. 1988), little is known regarding the contribution of these effectors to 5‐HT_1A_, GABA_B_, and GABA_A_ receptor‐mediated transmissions in the DR and whether manipulations of GIRK channels cause alterations in these pathways related to the pathophysiology of depression. Therefore, we sought to investigate in vitro, the contribution of GIRK2 subunit‐containing channels on the basal properties and tonic activity of DR neurons and their impact on 5‐HT_1A_, GABA_B_, and GABA_A_ receptor‐mediated transmission in the DR microcircuit.

## Methods

### Ethical approval

The animals used in this study were GIRK2 knockout mice (GIRK2 KO mice; *n* = 35) and their wild‐type littermates (WT; *n* = 45) (4–8 weeks old) derived from heterozygote crossing in a C57BL6/J background. Mice were maintained at 22 ± 2°C in a 12‐h light/dark cycle with food and water provided ad libitum. The genotype was verified by PCR screening of genomic DNA at the Department of Neurochemistry Research of the Basque Health Service (Zamudio, Spain). Every effort was made to minimize the animals' suffering and to minimize the number of animals used. All procedures were conducted in accordance with the European Community Council Directive on “The Protection of Animals Used for Experimental and Other Scientific Purposes” (2010/63/EU) and Spanish Law (RD 53/2013) for the care and use of laboratory animals. Experimental protocols were approved by the Local Committee for Animal Experimentation at the University of the Basque Country (permit numbers 44‐P04‐01/2010 and 44‐P04‐03/2010).

### Electrophysiological recordings

#### Slice preparation

Whole‐cell patch‐clamp recordings were performed as previously described (Gantz et al. 2015). Mice were anesthetized with isoflurane and killed via decapitation. Immediately afterward, the brains were removed and mounted in a vibratome chamber (Leica VT 1200S). The DR was located in slices that were rostral to the level where the aqueduct begins to meet the fourth ventricle and caudal to the decussation of the cerebellar peduncle. Coronal slices (220 μm) were prepared in ice‐cold artificial cerebrospinal fluid (ACSF, external bath solution) containing the following (in mmol/L): 126 NaCl, 2.5 KCl, 1.2 MgCl_2_, 2.6 CaCl_2_, 1.2 NaH_2_PO_4_, 11 D‐glucose, and 21.4 NaHCO_3_. ASCF was oxygenated and maintained at pH 7.4 by bubbling with 95% O_2_/5% CO_2_ gas. Slices were stored in glass vials at 34°C with oxygenated (95% O_2_/5% CO_2_) ACSF. To reduce neuronal damage, MK‐801 (10 μmol/L) was included in the cutting and initial incubation solution to block NMDA receptors. After an incubation period of at least 40 min, slices were transferred to the recording chamber for recording.

#### Electrophysiological recording

Slices containing DR were mounted on a recording chamber attached to a microscope (Eclipse FN1; Nikon) and continuously perfused with ACSF at 1.5‐3 mL/min at 32–34°C maintained using an in‐line solution heater (TC‐324; Warner Instruments, Hamden, CT). Only neurons that were located on the midline with a large cell body and that met the previously reported properties of tryptophan hydroxylase positive, non‐GABAergic DR neurons in mice, were included in this study (Gocho et al. 2013; Levitt et al. 2013).

Recording pipettes pulled from glass capillaries (1.7–3 MΩ; World Precision Instruments) were used for all the experiments. To record the passive and active membrane properties and 5‐CT‐ and baclofen‐induced currents, electrodes were filled with an internal solution containing the following (in mmol/L): 130 K‐gluconate, 5 NaCl, 1 MgCl_2_, 1 EGTA, 10 HEPES (K), 2 Mg‐ATP, 0.5 Na‐GTP, and 10 Na_2_‐phosphocreatine (pH: 7.3‐7.4, 280 mOsm). To record the spontaneous and evoked inhibitory postsynaptic currents (sIPSCs and eIPSCs), electrodes were filled with the following internal solution (in mmol/L): 70 K‐gluconate, 70 KCl, 2 NaCl, 4 EGTA, 10 HEPES (K), 4 Mg‐ATP, 0.3 Na‐GTP, and 10 Na_2_‐phosphocreatine (pH: 7.3–7.4, 280 mOsm). DNQX (10 μmol/L) was added to the external solution (ACSF) to block AMPA/kainate receptors. Cells were voltage‐clamped at −70 mV using a Multiclamp 700B amplifier (Molecular Devices (UK), Wokingham, UK). Signals were digitized using a Digidata 1440A (Molecular Devices) analog‐to‐digital converter. Data were acquired using pClamp 10 software (sampled at 10 kHz, filtered at 5 kHz) and were post hoc filtered (Molecular Devices). Series resistance was monitored without compensation with 5 mV depolarizing steps (200 msec) induced every 60 sec to ensure sufficient electrical access to the cell (<15 MΩ for inclusion).

Immediately after gaining access, the membrane capacitance, input resistance, and resting membrane potential (*I* = 0) were measured using the Clampex application of pClamp 10 software. Next, in current‐clamp mode, action potentials were elicited by applying currents from 0 to 200 pA in 50 pA steps (2 sec per step), and the action potential properties were determined. For the current–voltage (*I*/*V*) analysis, voltage steps were applied. Under this protocol, the presence of an inwardly rectifying K^+^ (IRK) current could be detected according to the typical current‐voltage relationship obtained when the membrane potential was hyperpolarized from −60 to −130 mV (−10 mV increments, 100 msec per step), as described by Williams et al. (1982). Activation of GIRK currents induced by 5‐CT was detected using the *I*/*V* protocol before and during drug application.

To study sIPSCs, the basal spontaneous activity of the cell was recorded for at least 3 min when the recording reached a steady and stable state. A paired‐pulse protocol was used to examine the eIPSCs. A monopolar stimulating electrode was positioned 100–200 μm dorsolateral to the cell being recorded, and pairs of electrical stimuli (100 μsec at 20 Hz, 100‐msec interpulse interval, 20‐sec interpair interval) were delivered using an Iso‐Flex stimulus isolator (A.M.P.I.). The liquid junction potential between the pipette internal solution and the ACSF was ~15 mV and was not corrected.

#### Electrophysiological data analysis

Analyses were carried out off‐line using the Clampfit application of pClamp 10 software. To calculate the effect of 5‐CT, baclofen, and GABA on the whole‐cell currents of the DR cells, the amplitude of the currents induced by these drugs was measured when the maximal effect was reached only in those cells where there was a complete washout after drug application.

A 30‐sec period was taken for the off‐line analysis of frequency, amplitude, rise time, and decay time of the sIPSCs for each experimental condition (before or during drug application). Single peak sIPSCs were detected using a semiautomated sliding template detection procedure (Clampfit 10.3 software). The template was generated by averaging multiple spontaneous currents, and the selection was fitted to four thresholds of the template. Each detected event was visually inspected and discarded if the amplitude was <7 pA.

The rise and decay times were determined by measuring the time between the currents crossing 10% and 90% of the baseline‐to‐peak amplitude range during the rise stage and decay stage of the event, respectively.

The amplitude of eIPSCs was calculated by measuring the average current at the peak of each evoked event and subtracted from the current obtained during the 10 ms window taken immediately before the stimulus artifact. To obtain the paired‐pulse ratio, the amplitudes of IPSC1 and IPSC2 were averaged for at least six sweeps (120 sec), and the ratio was calculated for each cell. By analyzing the paired‐pulse ratio, we detected if there was a paired‐pulse facilitation or depression pattern in each DR cell, which is related to changes in the probability of GABA release (Bonci and Williams 1997; Manzoni and Williams 1999).

### Drugs

The chemicals used to make the ACSF were purchased from Panreac or Merck. Mg‐ATP, Na‐GTP, and phosphocreatine disodium salt hydrate for the internal solution were obtained from Sigma‐Aldrich. Stock solutions of 5‐CT (5**‐**carboxamidotryptamine maleate salt; Sigma‐Aldrich, Madrid, Spain), baclofen [(*R*)‐4‐Amino‐3‐(4‐chlorophenyl)butanoic acid; Abcam, Cambridge, UK], CGP55845 (CGP55845 hydrochloride; Abcam, Cambridge, UK), GABA (4‐aminobutanoic acid; Abcam), tertiapin‐Q (Tocris, Bristol, UK), and WAY100635 (N‐[2‐[4‐(2‐methoxyphenyl)‐1‐piperazinyl]ethyl]‐N‐2‐pyridinylcyclohexanecarboxamide maleate salt; Sigma‐Aldrich) were prepared in distilled water. DNQX [6,7‐dinitroquinoxaline‐2,3‐dione; Abcam] and MK‐801 [(+)‐MK‐801 maleate; Abcam] were prepared in DMSO (dimethyl sulfoxide). Drug stocks were diluted in artificial cerebrospinal fluid (ACSF) immediately before application. The highest experimental concentration of DMSO was 0.01%. Isoflurane was purchased from Abbott.

### Statistics

Statistical analyses were performed using GraphPad Prism Software (v.5.01; GraphPad Software Inc., La Jolla, CA). Values are given as the means ± SEM. All data sets with *n* > 30 were tested for normality using a one‐sample Kolmogorov–Smirnov normality test. If any data set failed the normality test, nonparametric tests were used. The statistical analysis for within‐group comparisons was performed using paired two‐tailed *t*‐tests or a two‐way repeated measures (RM) ANOVA followed by Bonferroni multiple comparisons post hoc test. Between‐group significant differences were determined using unpaired two‐tailed *t*‐tests, two‐tailed Mann–Whitney *U* tests, or a two‐way ANOVA followed by Bonferroni multiple comparisons post hoc test. Fisher's exact test was used to compare the paired‐pulse ratio pattern. Correlations were determined using two‐tailed Pearson correlation. To determine whether the cumulative probability plots of the frequency and amplitude of sIPSCs were different between groups, the two‐sample Kolmogorov–Smirnov test was used. A difference of *P* < 0.05 was considered significant.

## Results

### Role of GIRK2 subunit‐containing GIRK channels in the electrophysiological properties of DR neurons

To examine the contribution of GIRK2 subunit‐containing GIRK channels on the electrophysiological properties of DR neurons, we inspected the intrinsic membrane properties and action potential (AP) properties in GIRK2 KO mice. As shown in Table 1, we observed a more depolarized resting membrane potential (*P* = 0.0318, Mann–Whitney *U* test) and a higher input resistance (*P* = 0.0113, unpaired two‐tailed *t*‐test) in GIRK2 KO mice compared to WT mice.

**Table 1:** General electrophysiological properties of *dorsal raphe* neurons of wild‐type and GIRK2 KO mice

	WT	GIRK2 KO
Intrinsic membrane properties		
Capacitance (pF)	52.29 ± 2.24 (23) (36.50–76.12)	52.33 ± 2.37 (38) (26.54–81.75)
Input resistance (MΩ)	479.40 ± 31.07 (24) (187.10–739.1)	667.00 ± 55.25* (35) (169.70–1599.15)
Resting potential (mV)	−57.71 ± 0.73 (56) (−)(73–50)	−55.58 ± 1.19# (43) (−)(79–40)
Action potential properties		
Threshold (mV)	−32.26 ± 1.94 (16) (−)(40.67–16.01)	−35.73 ± 0.97 (35) (−)(42.34–22.45)
Amplitude (mV)	71.48 ± 1.09 (17) (64.62–80.75)	72.70 ± 1.06 (35) (56.64–83.22)
Half‐width (msec)	1.55 ± 0.08 (17) (1.09–2.37)	1.33 ± 0.05* (36) (0.85–2.68)
Rise time (msec)	0.55 ± 0.03 (14) (0.38–0.71)	0.62 ± 0.03 (25) (0.15–0.84)
Decay time (msec)	1.42 ± 0.08 (17) (0.91–2.14)	1.16 ± 0.06* (36) (0.62–1.32)

Values represent the means ± SEM of (*n*) cells. *P *<* 0.05, unpaired two‐tailed *t*‐test; ^#^ *P* < 0.05, Mann–Whitney *U* test.

**Figure 1:**
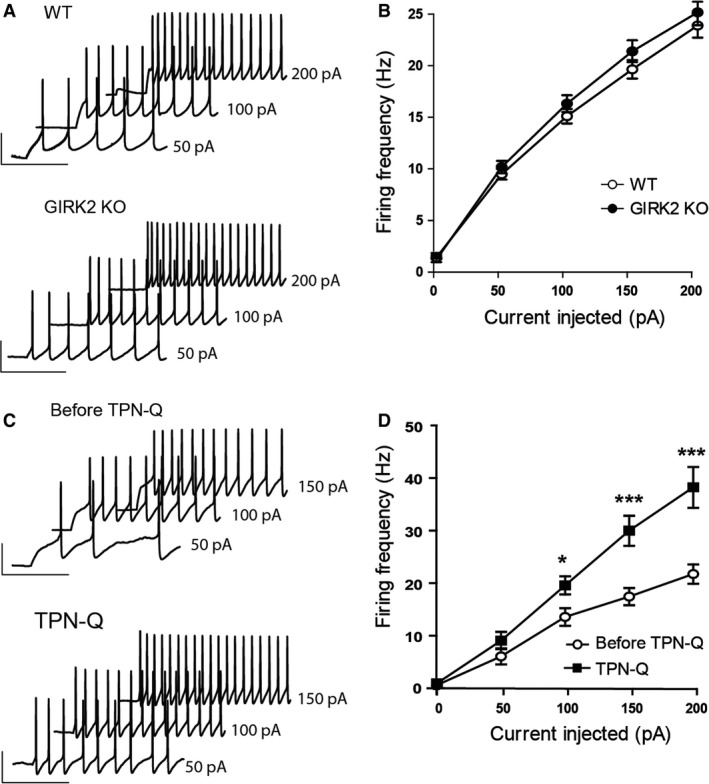
Electrophysiological properties of the action potentials of DR neurons from WT and GIRK2 KO mice. (A) Examples of action potential (AP) waveforms of DR neurons from the WT (left) and GIRK2 KO mice (right) elicited by current injections of 50, 100, and 200 pA. Scale bars, 40 pA, 200 msec. (B) Input‐output relationship curves of DR neurons from WT (*n* = 24 cells) and GIRK2 KO mice (*n* = 35 cells). (C) Examples of AP waveforms of DR neurons from the WT before (Before TPN‐Q; left) and during tertiapin‐Q bath application (TPN‐Q; 1 μmol/L; right) elicited by current injections of 50, 100, and 150 pA. Scale bars, 40 pA, 200 msec. (D) Input‐output relationship curves of DR neurons from the WT mice before and during tertiapin‐Q application (*n* = 11 cells; *P < 0.05 and ***P < 0.001, Bonferroni post hoc test). Means ± SEM.

In current‐clamp mode, injection of current produced slow and regular firing of long‐duration AP that increased in frequency upon larger current injections in both genotypes (*F*_1,228_ = 558.6, *P* < 0.0001, two‐way RM ANOVA; Fig. 1A). As shown in Figure 1B, the genotype had no effect on the sensitivity of DR neurons to the injected currents (*F*_1,228_ = 0.8321, *P* = 0.3655, two‐way RM ANOVA). Analysis of the AP properties revealed that the DR neurons of the GIRK2 KO mice had shorter AP half‐width (*P* = 0.0344, unpaired two‐tailed *t*‐test; Table 1) and AP decay time (*P* = 0.0175, unpaired two‐tailed *t*‐test; Table 1). Similarly, the application of the GIRK channel blocker tertiapin‐Q (1 μmol/L) during 5 min in WT mice depolarized the resting membrane potential (*P* < 0.0001, paired two‐tailed *t*‐test; *n* = 19 cells), reduced the AP decay time (*P* = 0.0115, paired two‐tailed *t*‐test; *n* = 11 cells), and increased the AP rise time (*P* = 0.0219, paired two‐tailed *t*‐test; *n* = 11 cells) without altering the AP half‐width (*P* = 0.3849, paired two‐tailed *t*‐test; *n* = 11 cells). Moreover, tertiapin‐Q increased the sensitivity of DR neurons to the injected currents (*F*_1,77_ = 49.18, *P* < 0.0001, two‐way ANOVA; *n* = 11 cells; Fig. 1C and D), being the AP frequency after 100, 150, and 200 pA current injections statistically greater after the application of tertiapin‐Q (*P* < 0.05 for 100 pA current injections and *P* < 0.001 for 150 and 200 pA current injections, Bonferroni post hoc test; *n* = 11 cells). In summary, these results indicate that both the genetic or pharmacological blockade of GIRK channels depolarizes the membrane potential. In addition, the genetic inactivation of GIRK channels increases the membrane resistance and reduces the duration of action potentials of DR neurons.

### Decreased 5‐HT_1A_ and GABA_B_ receptor‐mediated postsynaptic currents in DR neurons of GIRK2 KO mice

**Figure 2:**
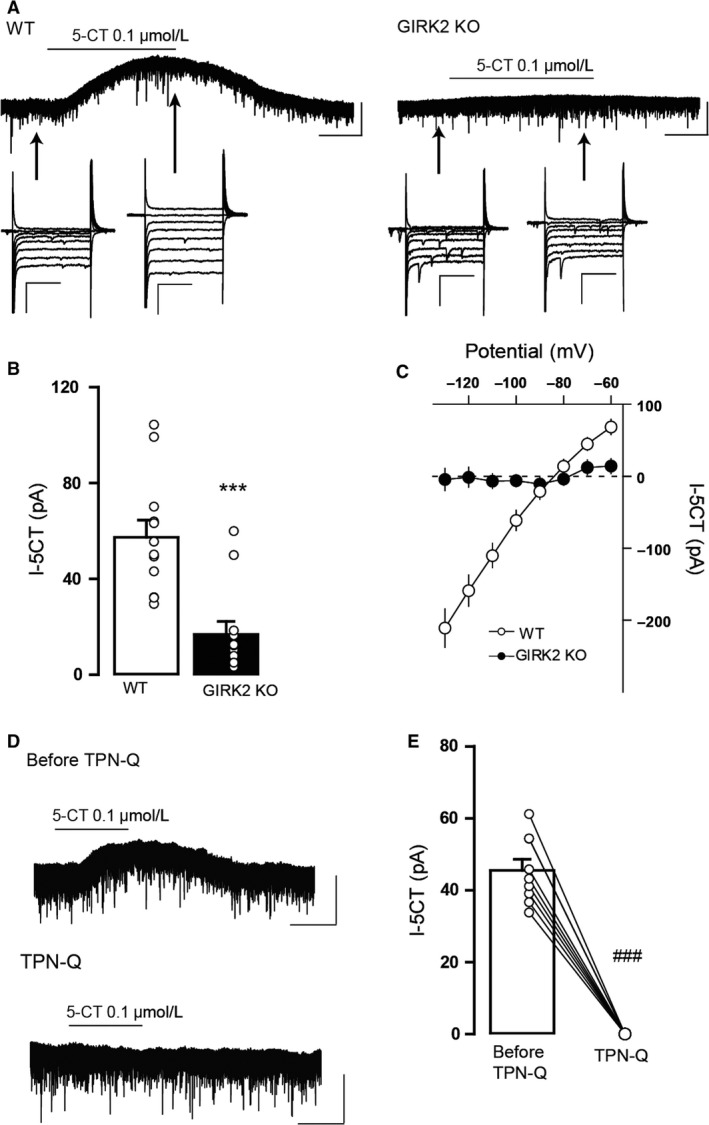
5‐CT‐induced outward currents in DR neurons from WT and GIRK2 KO mice. (A) Representative whole‐cell recordings of the outward current induced by 5‐CT (I‐5‐CT; 0.1 μmol/L) for the WT (left) and GIRK2 KO mice (right). Scale bars, 50 pA, 60 sec. Voltage steps (−60 to −130 mV) were performed before and during 5‐CT bath application. Scale bars, 200 pA, 50 msec. (B) Summary bar graph showing the amplitude of the current induced by 5‐CT (0.1 μmol/L) in the WT and GIRK2 KO mice (*n* = 12–13 cells per group; ***P < 0.001, unpaired two‐tailed *t*‐test). (C) Summary current‐voltage plot of I‐5‐CT versus potential for WT and GIRK2 KO mice. Notice the absence of inward rectification in the GIRK2 KO mice. (D) Representative recordings of the outward current induced by 5‐CT (0.1 μmol/L) in the WT mice before (Before TPN‐Q) and during (TPN‐Q) tertiapin‐Q (1 μmol/L). Scale bars, 50 pA, 60 sec. (E) Summary bar graph showing the amplitude of the current induced by 5‐CT (0.1 μmol/L) in WT before and during tertiapin (1 μmol/L) bath application (*n* = 9 cells; ^###^ *P* < 0.0001, paired two‐tailed *t*‐test). Bars represent the means ± SEM. In B and E, values from a single experiment are denoted by circles.

To evaluate whether the 5‐HT_1A_ receptor‐mediated postsynaptic transmission in DR neurons is affected by *Girk2* gene inactivation, we measured the somatodendritic currents evoked by 5‐CT, a high‐affinity full agonist of 5‐HT_1A_ receptors, in DR cells of GIRK2 KO mice. Whole‐cell voltage‐clamp recordings performed in WT mice revealed that the application of 5‐CT (0.1 μmol/L) elicited outward currents that were markedly reduced in the GIRK2 KO mice (*P* = 0.0004, unpaired two‐tailed *t*‐test; Fig. 2A and B). Voltage steps (−60 to −130 mV, 100 msec) were applied to determine the identity of the 5‐CT‐induced outward currents before and during drug application. The current produced by 5‐CT at each voltage was determined by subtracting the corresponding control current at baseline conditions, before the bath application of 5‐CT. Consistent with the activation of GIRK channels, the 5‐CT‐induced current displayed inward rectification in WT but not in GIRK2 KO animals. This is shown as the change in the slope of the current‐voltage relationship, which was more pronounced at more hyperpolarized potentials (Fig. 2A and C). Moreover, application of tertiapin‐Q (1 μmol/L) before 5‐CT completely blocked the outward currents induced by this drug in the WT mice (*P* < 0.0001, paired two‐tailed *t*‐test; Fig. 2D and E).

**Figure 3:**
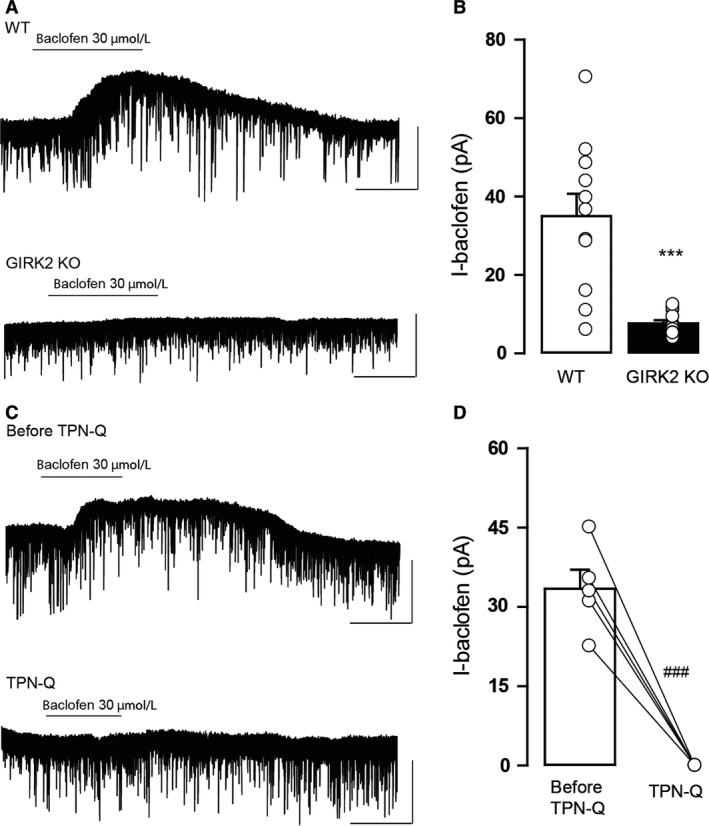
Baclofen‐induced outward currents in DR neurons from WT and GIRK2 KO mice. (A) Representative whole‐cell recordings of the outward current induced by baclofen (I‐Baclofen; 30 μmol/L) for the WT and GIRK2 KO mice. Scale bars, 50 pA, 60 sec. (B) Summary bar graph showing the amplitude of the current induced by baclofen (30 μmol/L) for WT and GIRK2 KO mice (*n* = 11–13 cells per group; ***P < 0.0001, unpaired two‐tailed *t*‐test). (C) Representative recordings of the outward current induced by baclofen (30 μmol/L) in the WT mice before (Before TPN‐Q) and during (TPN‐Q) tertiapin‐Q (1 μmol/L). Scale bars, 50 pA, 60 sec. (D) Summary bar graph showing the amplitude of the current induced by baclofen (30 μmol/L) in WT before and during tertiapin‐Q (1 μmol/L) bath application (*n* = 5 cells; ^###^ *P* < 0.001, paired two‐tailed *t*‐test) Bars represent the means ± SEM. Values from a single experiment are denoted by circles.

In addition to 5‐HT_1A_ receptors, GIRK channels are coupled to GABA_B_ receptors in the DR. Thus, in the second set of experiments, we studied the effect of *Girk2* gene ablation on the somatodendritic currents induced by the GABA_B_ receptor agonist baclofen. Application of baclofen (30 μmol/L) elicited outward currents in WT that were statistically reduced in the GIRK2 KO mice (*P* < 0.0001, unpaired two‐tailed *t*‐test; Fig. 3A and B). Similarly, the baclofen‐induced currents were completely absent when tertiapin‐Q was previously applied in WT mice (*P* = 0.0008, paired two‐tailed *t*‐test; Fig. 3C and D). Taken together, these data indicate that GIRK channels, and specifically those containing GIRK2 subunits control the 5‐HT_1A_ and GABA_B_ receptor‐mediated postsynaptic currents in the DR.

### Decreased GABA release in GIRK2 KO mice

**Figure 4:**
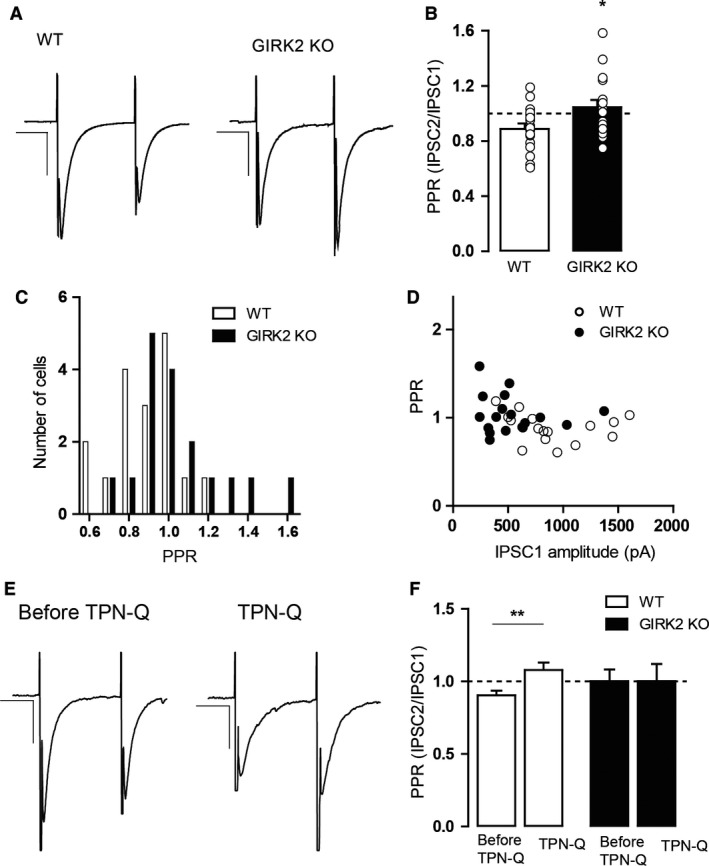
Characterization of the paired‐pulse ratio of eIPSCs in DR neurons from WT and GIRK2 KO mice. (A) Examples of paired‐pulse traces from recordings of eIPSCs for the WT (left) and GIRK2 KO mice (right). IPSCs from WT showed paired‐pulse depression, unlike those from the GIRK2 KO mice, which showed paired‐pulse facilitation. Scale bars, 400 pA, 20 msec for WT and 200 pA, 20 msec for GIRK2 KO. (B) Summary histograms showing the paired‐pulse ratio (PPR) for WT and GIRK2 KO mice. (*n* = 17 cells per group; *P < 0.05, unpaired two‐tailed *t*‐test). (C) Cumulative distribution of the PPR for all the recorded neurons from WT and GIRK2 KO mice (*n* = 17 cells per group). (D) PPR is independent of the amplitude of IPSC1 for both genotypes (*n* = 17 cells per group). (E) Examples of paired‐pulse traces from recordings of eIPSCs for the WT mice before (left) and during (right) tertiapin‐Q (TPN‐Q; 1 μmol/L) bath application. Scale bars, 200 pA, 20 msec. (F) Summary histograms showing the paired‐pulse ratio (PPR) before and during TPN‐Q for WT mice (*n* = 13 cells; *P < 0.01, paired two‐tailed *t*‐test) and GIRK2 KO mice (*n* = 17 cells). Bars represent the means ± SEM. Values from a single experiment are denoted by circles.

Next, we examined whether *Girk2* gene inactivation alters presynaptic and postsynaptic GABA_A_ receptor‐mediated neurotransmission in the DR. To study the presynaptic GABA_A_ receptor activity, we assessed GABA release in GIRK2 KO mice using two well‐established methods. The first method was the paired‐pulse stimulation protocol. In synapses with low release probability, the second release is enhanced due to residual calcium in the presynaptic terminal, and changes in neurotransmitter release affect the paired‐pulse ratio (Khazipov et al. 1995; Bonci and Williams 1997; Stanford and Cooper 1999; Sullivan 1999; Melis et al. 2002). As shown in Figure 4A, in cells from WT mice, the second eIPSC (IPSC2) was generally smaller than the first eIPSC (IPSC1), indicating depressing synapses, and cells from GIRK2 KO displayed a larger IPSC2 compared to IPSC1, which is characteristic of facilitating synapses with low probability of release. Thus, the proportion of cells that exhibited depressing synapses was significantly reduced in the GIRK2 KO mice (*P* < 0.0001, Fisher's exact test), and the paired‐pulse ratio in the GIRK2 KO mice was larger than in WT mice (*P* = 0.0223, unpaired two‐tailed *t*‐test; Fig. 4B). The numbers of cells exhibiting facilitation and depression are illustrated in Figure 4C. Importantly, the paired‐pulse ratio did not depend on the size of IPSC1 for any genotype (*P* = 0.4049 for WT, *P* = 0.5849 for GIRK2 KO, two‐tailed Pearson correlation; Fig. 4D). Additionally, the effect of tertiapin‐Q on the paired‐pulse ratio was inspected. As shown in Figure 4E and F, application of tertiapin‐Q increased the paired‐pulse ratio in WT mice (*P* = 0.002, paired two‐tailed *t*‐test) but not in the GIRK2 KO mice (*P* = 0.9855, paired two‐tailed *t*‐test).

**Figure 5:**
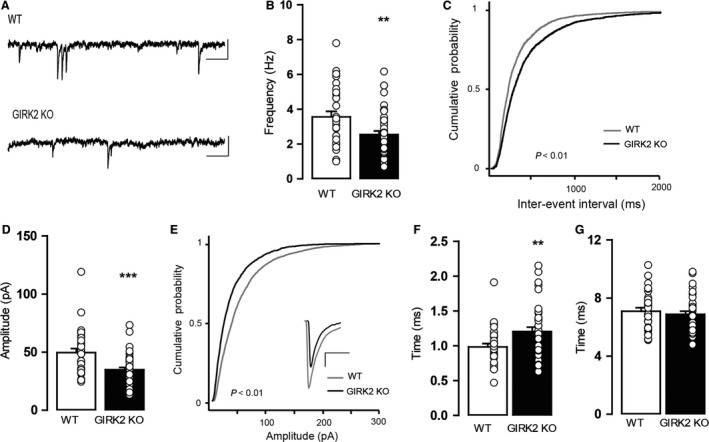
Characterization of GABAergic sIPSCs in DR neurons from WT and GIRK2 KO mice. (A) Representative current traces showing sIPSCs for the WT and GIRK2 KO mice. Scale bars, 50 pA, 100 msec. (B) Summary bar graph showing the frequency of sIPSCs in both groups (*n* = 31–44 cells per group; **P < 0.01, unpaired two‐tailed *t*‐test). (C) Cumulative probability histograms of interevent intervals of sIPSCs in WT (*n* = 3437 events from 31 cells) and GIRK2 KO mice (*n* = 2919 events from 44 cells). (D) Summary bar graph showing the amplitude of sIPSCs for both groups (*n* = 31–44 cells per group; ***P < 0.0001, two‐tailed Mann–Whitney *U* test). (E) Cumulative probability histograms of the amplitudes of sIPSCs for the WT (*n* = 3517 events from 31 cells) and GIRK2 KO mice (*n* = 2987 events from 44 cells). Inset shows an enlarged depiction of the averaged individual events in the WT and GIRK2 KO mice. Scale bars, 10 pA, 5 msec. (F) Summary bar graph showing the rise time of sIPSCs for the WT and GIRK2 KO mice (*n* = 31–36 cells per group; **P < 0.01, unpaired two‐tailed *t*‐test). (G) Summary bar graph showing the decay time of sIPSCs for the WT and GIRK2 KO mice (*n* = 31–36 cells per group). Bars represent the means ± SEM. Values from a single experiment are denoted by circles.

The second method that we used to examine GABA release in the GIRK2 KO mice was assessing sIPSC frequency. The frequency of the sIPSCs of the GIRK2 KO mice was significantly lower than the sIPSCs of WT mice (*P* = 0.0062, unpaired two‐tailed *t*‐test; Fig. 5A and B). Thus, the cumulative probability plot of the interevent interval was shifted significantly to the right for the GIRK2 KO mice (*P* < 0.01, K–S test; Fig. 5C). In addition, the sIPSC amplitude of the GIRK2 KO mice exhibited a decrease compared to that of the WT mice (*P* = 0.0001, two‐tailed Mann–Whitney *U* test; Fig. 5D). This reduction was confirmed by a shift to the left in the cumulative probability plot of amplitudes (*P* < 0.01, K–S test; Fig. 5E). The analysis of the kinetic parameters revealed that the rise time was greater in the GIRK2 KO compared to WT mice (*P* = 0.01, unpaired two‐tailed *t*‐test; Fig. 5F) but the decay time was not different (*P* = 0.5212, unpaired two‐tailed *t*‐test; Fig. 5G).

These results demonstrate that GIRK2 KO mice have a decreased spontaneous probability of GABA release and a reduced number and/or sensitivity of postsynaptic GABA_A_ receptors. Moreover, both the genetic and pharmacological blockade of GIRK channels cause a decrease in the evoked probability of GABA release.

### Diminished control of presynaptic 5‐HT_1A_ and GABA_B_ receptors over GABA release in GIRK2 KO mice

One physiological function of inhibitory G protein‐coupled receptors (GPCRs), which activate GIRK channels, is the inhibition of presynaptic neurotransmitter release (Thompson et al. 1993; Wu and Saggau, 1997). Therefore, we examined whether the presynaptic inhibition of GABA release was altered in GIRK2 KO mice.

**Figure 6:**
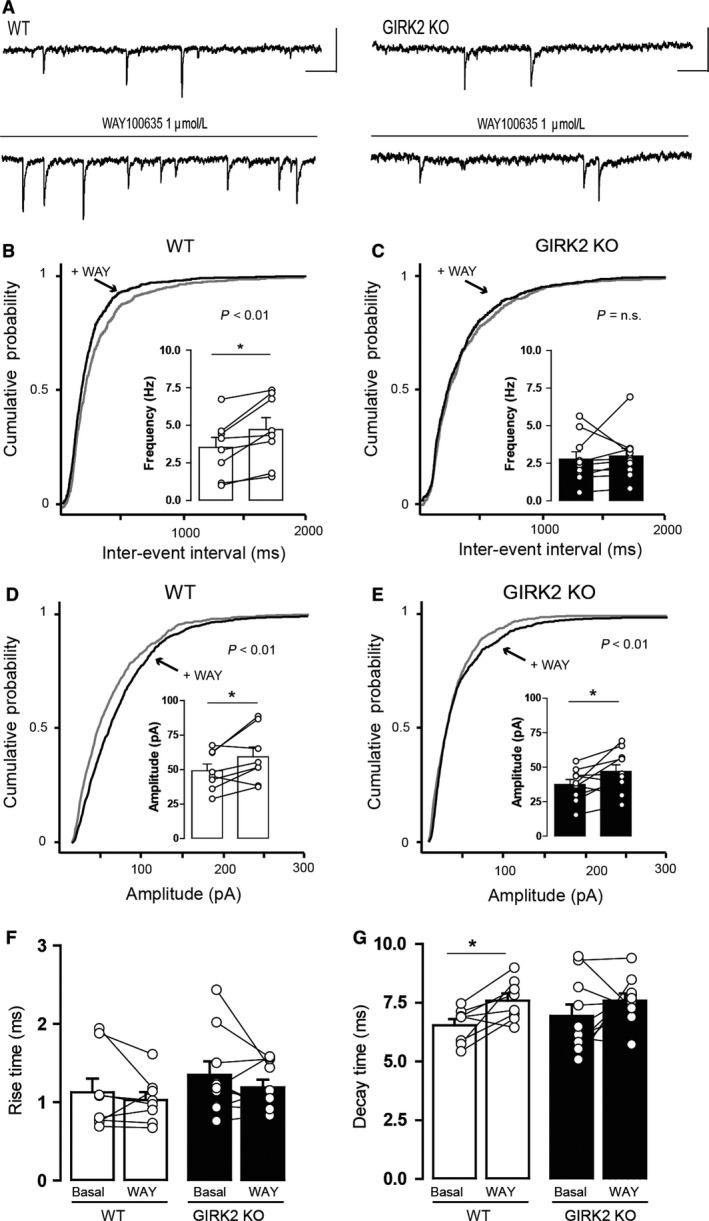
Effect of the 5‐HT_1A_ receptor antagonist WAY100635 on the sIPSCs of DR neurons from WT and GIRK2 KO mice. (A) Representative current traces before and during 1 μmol/L WAY100635 (WAY) application for the WT (left) and GIRK2 KO mice (right). Scale bars, 50 pA, 100 msec. (B‐C) Cumulative probability histograms showing that WAY100635 induced a significant shift in the distribution of interevent intervals in (B) the WT (*n* = 843–1139 events from eight cells) but not in (C) the GIRK2 KO mice (*n* = 844–881 events from 10 cells). Insets display summary histograms showing that WAY100635 increased the average frequency of sIPSCs in WT but not in GIRK2 KO mice (*n* = 8–10 cells per group; *P < 0.05, paired two‐tailed *t*‐test). (D–E) Cumulative probability histograms showing that WAY100635 induced a significant shift in the distribution of amplitudes in (D) WT (*n* = 853–1141 events from eight cells) and in (E) GIRK2 KO mice (*n* = 845–906 events from 10 cells)_._ Insets display summary histograms showing that WAY100635 increased the average amplitude of sIPSCs in both genotypes (*n* = 8–10 cells per group; *P < 0.05, paired two‐tailed *t*‐test). (F–G) Summary bar graph showing the effect of WAY100635 application on (F) the rise time and (G) the decay time of sIPSCs for the WT and GIRK2 KO mice (*n* = 8–10 cells per group; *P < 0.05, paired two‐tailed *t*‐test). Bars represent the means ± SEM. Values from a single experiment are denoted by circles.

In the first set of experiments, we evaluated the control of presynaptic 5‐HT_1A_ receptors over GABA release in the DR neurons of GIRK2 KO mice. WAY100635 was perfused (1 μmol/L, 10 min), and the frequency of sIPSCs was inspected. In addition, we studied the amplitude, rise time, and decay time of sIPSCs before and during the application (Fig. 6A).

WAY100635 increased the frequency of sIPSCs in WT mice (*P* = 0.0094, paired two‐tailed *t*‐test; Fig. 6B, inset histogram) but not in GIRK2 KO mice (*P* = 0.6748, paired two‐tailed *t*‐test; Fig. 6C, inset histogram). This effect is reflected as a significant shift to the left of the cumulative probability plot of the interevent interval for WT mice (*P* < 0.01, K–S test; Fig. 6B) but not for GIRK2 KO mice (*P* = 0.7223, K–S test; Fig. 6C). Furthermore, WAY100635 increased the amplitude of sIPSCs in both WT (*P* = 0.0420, paired two‐tailed *t*‐test; Fig. 6D, inset histogram) and GIRK2 KO mice (*P* = 0.0294, paired two‐tailed *t*‐test; Fig. 6E, inset histogram), represented as a shift to the right in the cumulative probability plot of amplitudes for WT mice (*P* < 0.01, K–S test; Fig. 6D) and for GIRK2 KO mice (*P* = 0.01, K–S test; Fig. 6E). The analysis of the effect of WAY100635 on the rise time of sIPSCs revealed that it had no effect on both WT (*P* = 0.4293, paired two‐tailed *t*‐test; Fig. 6F) and GIRK2 KO mice (*P* = 0.2674, paired two‐tailed *t*‐test; Fig. 6F). In the WT mice, WAY100635 induced a significant increase in decay time (*P* = 0.0145, paired two‐tailed *t*‐test; Fig. 6G) that was not observed in the GIRK2 KO mice (*P* = 0.2356, paired two‐tailed *t*‐test; Fig. 6G).

**Figure 7:**
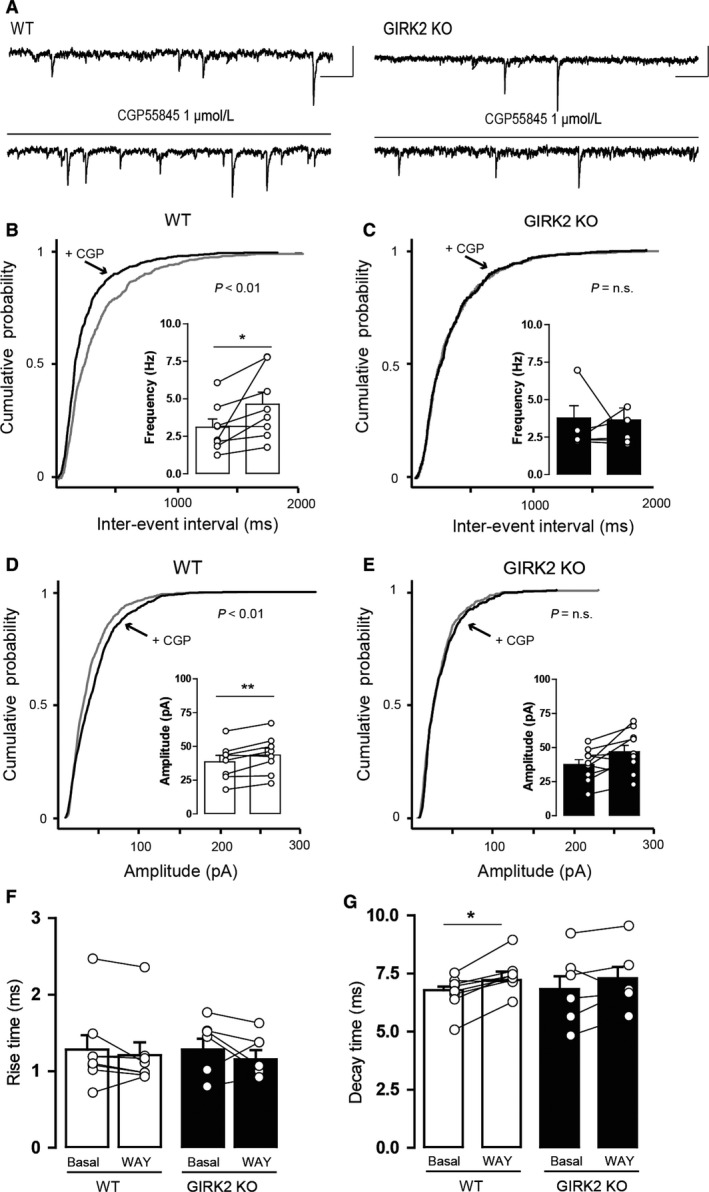
Effect of the GABA_B_ receptor antagonist CGP55845 on the sIPSCs of DR neurons from WT and GIRK2 KO mice. (A) Representative current traces before and during 1 μmol/L CGP55845 (CGP) application in the WT (left) and GIRK2 KO mice (right). Scale bars, 50 pA, 100 msec. (B–C) Cumulative probability histograms showing that CGP55845 induced a significant shift in the distribution of interevent intervals in (B) WT (*n* = 748–1108 events from eight cells) but not in (C) GIRK2 KO mice (*n* = 528–584 events from six cells). Insets display summary histograms showing that CGP55845 increased the average frequency of sIPSCs in WT but not in GIRK2 KO mice (*n* = 6–8 cells per group; *P < 0.05, paired two‐tailed *t*‐test). (D–E) Cumulative probability histograms showing that CGP55845 induced a significant shift in the distribution of amplitudes in (D) WT (*n* = 753–1115 events from eight cells) but not in (E) GIRK2 KO mice (*n* = 536–595 events from six cells)_._ Insets display summary histograms showing that CGP55845 increased the average amplitude of sIPSCs in WT but not in GIRK2 KO mice (*n* = 6–8 cells per group; **P < 0.01, paired two‐tailed *t*‐test). (F–G) Summary bar graph showing the effect of CGP55845 application on (F) the rise time and (G) the decay time of sIPSCs in the WT and GIRK2 KO mice (*n* = 6–8 cells per group; *P < 0.05, paired two‐tailed *t*‐test). Bars represent the means ± SEM. Values from a single experiment are denoted by circles.

In the second group of experiments, we inspected the presynaptic inhibition of GABA_B_ receptors in the GIRK2 KO mice. For this purpose, CGP55845 was perfused (1 μmol/L, 10 min), and the sIPSC parameters were examined before and during drug application (Fig. 7A). CGP55845 caused an increase in the frequency of sIPSCs in the WT mice (*P* = 0.0431, paired two‐tailed *t*‐test; Fig. 7B, inset histogram) but not in the GIRK2 KO mice (*P* = 0.7461, paired two‐tailed *t*‐test; Fig. 7C, inset histogram). This effect is illustrated as a significant shift to the left of the cumulative probability plot of the interevent interval for the WT mice (*P* < 0.01, K–S test; Fig. 7B) but not for the GIRK2 KO mice (*P* = 0.5644, K–S test; Fig. 7C). CGP55845 increased the amplitude of sIPSCs in the WT mice (*P* = 0.0053, paired two‐tailed *t*‐test; Fig. 7D, inset histogram) but not in the GIRK2 KO mice (*P* = 0.1015, paired two‐tailed *t*‐test; Fig. 7E, inset histogram). This change was represented as a shift to the right in the cumulative probability plot of amplitudes for the WT mice (*P* < 0.01, K–S test; Fig. 7D) but not for the GIRK2 KO mice (*P* = 0.1285, K–S test; Fig. 7E). No effect of the drug was observed in the rise time of the events in the WT mice (*P* = 0.2514, paired two‐tailed *t*‐test; Fig. 7F) or in the GIRK2 KO mice (*P* = 0.4045, paired two‐tailed *t*‐test; Fig. 7F). However, CGP55845 increased the decay time of the sIPSCs in the WT mice (*P* = 0.0179, paired two‐tailed *t*‐test; Fig. 7G) but not in the GIRK2 KO mice (*P* = 0.2895, paired two‐tailed *t*‐test; Fig. 7G).

These results indicate that the control of GABA release exerted by presynaptic 5‐HT_1A_ and GABA_B_ receptors is impaired in GIRK2 KO mice.

### Reduced postsynaptic GABA activity in GIRK2 KO mice

**Figure 8:**
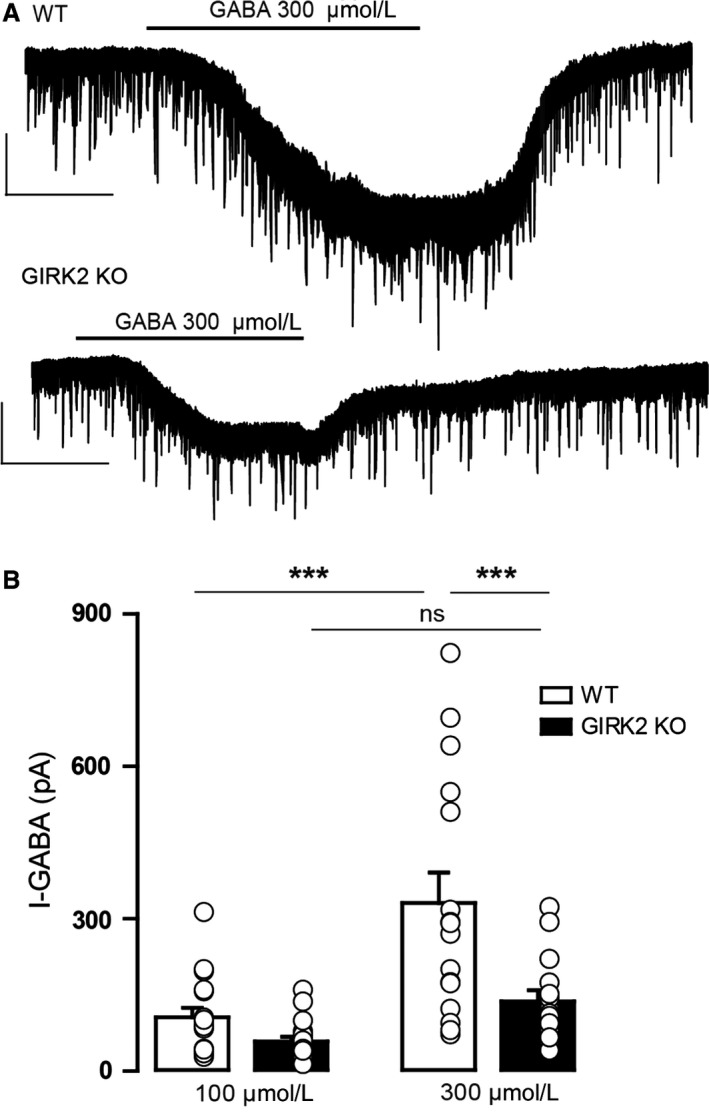
GABA‐induced inward currents in DR neurons from WT and GIRK2 KO mice. (A) Representative whole‐cell recordings of the outward currents induced by GABA (I‐GABA; 300 μmol/L) for the WT and GIRK2 KO mice. Scale bars, 100 pA, 60 sec. (B) Summary bar graph showing the amplitude of the current induced by GABA (100 and 300 μmol/L) in the WT and GIRK2 KO mice (*n* = 15–18 cells per group; ***P < 0.001, Bonferroni post hoc test). Bars represent the means ± SEM. Values from a single experiment are denoted by circles.

Finally, we examined whether *Girk2* gene inactivation alters postsynaptic GABA_A_ receptor‐mediated neurotransmission in the DR. We inspected the whole‐cell currents of DR neurons induced by two different concentrations of GABA (100 and 300 μmol/L) in the GIRK2 KO mice. In both genotypes, GABA elicited fast‐onset inward currents that returned to baseline upon GABA washout (Fig. 8A). Nevertheless, a two‐way ANOVA revealed that there was a significant effect of genotype on the effect induced by GABA (*F*_1,64_ = 10.55, *P* = 0.0018), as the effect induced by GABA 300 μmol/L was greater in the WT mice than in the GIRK2 KO mice (*P* < 0.001, Bonferroni post hoc test; Fig. 8B). Moreover, the effect of GABA was concentration‐dependent in the WT mice but not in the GIRK2 KO mice (F_1,64_ = 15.75, *P* < 0.001, two‐way RM ANOVA followed by Bonferroni post hoc test; Fig. 8B). These results demonstrate that *Girk2* gene deletion reduces the number, activity, or time open of postsynaptic GABA_A_ receptors.

## Discussion

This study shows that GIRK2 subunits of GIRK channels contribute to the basal electrophysiological properties of DR neurons and play a pivotal role in 5‐HT_1A_ and GABA_B_ receptor‐mediated postsynaptic currents. Moreover, the fast GABA synaptic activity and the 5‐HT_1A_ and GABA_B_ receptor‐mediated presynaptic inhibition of GABA release is affected by inactivation of the *Girk2* gene.

Genetic deletion of the GIRK2 subunit decreased the resting membrane potential, effect that was mimicked by the pharmacological blockade of GIRK channels, and increased the membrane input resistance of DR neurons. These findings demonstrate that there is a basal GIRK conductance that contributes to setting the basal membrane properties of DR neurons. In this sense, the more depolarized resting membrane potential, likely due to the relatively smaller resting K^+^ conductance, may lead to a higher membrane input resistance in DR neurons. The GIRK2 subunit is the predominant subunit in neuronal GIRK channels and is responsible for the generation of GIRK currents in several brain regions (Liao et al. 1996; Luscher et al. 1997; Cruz et al. 2008; Torrecilla et al. 2008). Various studies have demonstrated that GIRK2 subunits primarily hyperpolarize the membrane potential of LC and hippocampal neurons (Luscher et al. 1997; Torrecilla et al. 2002), but others have provided negative results pertaining to layer 5/6 of the prelimbic cortex (Hearing et al. 2013). Although there is evidence that GIRK2 subunit expression levels in the DR are lower than those of GIRK1 and GIRK3 (Fairchild et al. 2003; Saenz del Burgo et al. 2008), our results demonstrate that, irrespective of expression levels, GIRK channels containing GIRK2 subunits control the electrophysiological properties of DR neurons. Present results also indicate that GIRK2 subunit‐containing channels modulate, somehow, the duration of AP, since GIRK2 KO mice showed shorter half‐width and decay times. Of note, these results were not fully replicated by the application of tertiapin‐Q in WT mice; although tertiapin‐Q reduced the decay time, it also increased the rise time resulting in action potentials of unaltered duration. To the best of our knowledge, although K^+^ conductance is involved in the generation of action potentials (Hille 2001), it is unlikely that GIRK channels directly modulate the firing properties of action potentials, as it is demonstrated by our further experiments where tertiapin‐Q did not mimic the changes in AP observed in GIRK2 KO mice. Therefore, the alterations in AP properties could be a consequence of the reduced GABAergic synaptic activity that the *Girk2* deletion causes. In fact, modifications of the synaptic activity (e.g., blockade of GABA_A_ receptors) were demonstrated to modulate properties of AP of layer 2/3pyramidal neurons causing a shift from afterhyperpolarization to afterdepolarization and increased the input resistance (Imbrosci and Mittmann 2013). In addition, tertiapin‐Q produced an increase in the firing rate upon current injections and this effect was not seen in GIRK2 KO mice. It is likely that this altered input‐output function observed after the pharmacological, but not genetic inhibition of GIRK channels is due to the complete blockade of GIRK channels and not just GIRK2 subunit‐containing channels.

We showed that 5‐CT‐ and baclofen‐induced whole‐cell postsynaptic currents were greatly reduced in the DR neurons of GIRK2 KO mice and completely prevented by the preapplication of tertiapin‐Q in WT mice. These findings strongly suggest that the activation of GIRK2 subunit‐containing channels is necessary to mediate the postsynaptic effect of 5‐HT_1A_ and GABA_B_ receptor activation in DR neurons. It is known that in vitro, 5‐HT_1A_ receptors in the mouse DR desensitize when activated by acute (15 min to 1 h) application of 5‐HT_1A_ receptor agonists (Riad et al. 2001; Beck et al. 2004; Bouaziz et al. 2014). As a result, repeated application of 5‐HT_1A_ and GABA_B_ receptor agonist onto DR slices would result in currents of smaller magnitudes, precluding the construction of concentration‐response curves in individual cells. Desensitization, however, could not have confounded the results obtained after the application of tertiapin‐Q since there was a complete absence of 5‐CT and baclofen‐induced currents, indicating that GIRK2 subunit‐containing channels control 5‐HT_1A_ and GABA_B_ receptor‐mediated responses in the DR. Although not studied in the DR until now, the involvement of GIRK2 subunits in whole‐cell postsynaptic currents induced by the stimulation of GPCRs, such as A1 adenosine receptors, GABA_B_ receptors, 5‐HT_1A_ receptors, and *μ* receptors, has been demonstrated in various brain areas, including the hippocampus, the VTA, and the LC (Luscher et al. 1997; Torrecilla et al. 2002; Cruz et al. 2008; Arora et al. 2010). In contrast, a recent pharmacological study of GIRK channels in 5‐HT neurons claimed that 5‐HT_1A_ receptor‐coupled GIRK channels may not be formed by GIRK1‐GIRK2 heteromers (Montalbano et al. 2015), which is the predominant form found in neurons (Liao et al. 1996). Regardless of the precise GIRK composition in the DR, our study provides direct evidence supporting the principal role of the GIRK2 subunit in mediating postsynaptic inhibition induced by GPCRs.

One key finding is that GIRK inactivation decreases the spontaneous and evoked probability of GABA release and it is supported by three distinct electrophysiological results. First, we observed that in GIRK2 KO mice the paired‐pulse ratio was greater compared to WT mice, effect that is associated with decreased neurotransmitter release (Bonci and Williams 1997; Dobrunz and Stevens 1997; Melis et al. 2002). Second, the application of tertiapin‐Q produced an increment in the paired‐pulse ratio in WT mice, but not in GIRK 2 KO mice. Third, the frequency of the sIPSCs of DR neurons was reduced in the GIRK2KO mice, which is indicative of presynaptic regulation of release (Van der Kloot 1991). Since the reduced frequency of sIPSC demonstrates a corresponding decreased amplitude of sIPSC, it could be arguable whether the change in frequency in GIRK2 KO is due to an increased number of undetected events hidden in the noise, indicating an effect of GIRK exclusively in the postsynaptic site. Nevertheless, the increased paired‐pulse ratio in GIRK2 KO mice and in WT mice after the application of tertiapin‐Q supports a presynaptic role of GIRK channels in GABA release. On one hand, the reduced probability of GABA release could be indicative of a decrease in the readily releasable vesicles (Dobrunz and Stevens 1997) or alterations in the cAMP‐dependent modulation of GABA release (Bonci and Williams, 1997 ; Cameron and Williams, 1993). On the other hand, the impaired GABA release could be consequence of an altered presynaptic inhibition. In this sense, it is not likely that GIRK inactivation directly impacts on the intracellular processes related to the regulation of synaptic release, but rather impairs the presynaptic inhibition. Importantly, if GIRK blockade were affecting exclusively the presynaptic GABA_B_ receptor activity, GABA release should be increased. Nevertheless, GABA release is not only regulated by presynaptic GABA_B_ receptors which reduce the release of GABA (Brenowitz et al. 1998; Waldmeier et al. 2008; Kobayashi et al. 2012), but also by presynaptic GABA_A_ receptors, which enhance vesicle release (Trigo et al. 2008; Howell and Pugh 2016). Both GABA_A_ and GABA_B_ receptors are coexpressed in a wide range of terminals including afferents to the DR (Soiza‐Reilly et al. 2013) and their coactivation modulates synaptic transmission (Howell and Pugh 2016). Interestingly, the GABA_B_ receptor activation regulates the membrane expression of GABA_A_ receptors through a BDNF‐dependent signaling pathway (Kuczewski et al. 2011). These facts together with our findings suggest that there may be some kind of presynaptic GABA_A_‐GABA_B_ interactions in which the lack of function of GABA_B_ receptors weakens the GABA_A_ receptor activity so that the net effect of GIRK inactivation is a reduction in GABA release. Future studies in this direction could elucidate the exact mechanism by which GIRK channels modulate GABA release.

Consistent with previous findings indicating that GABA_A/B_ and 5‐HT1_A/B_ agonists decrease 5‐HT and GABA release in the DR (Bagdy et al. 2000), present results show that in WT mice, presynaptic 5‐HT1_A_ and GABA_B_ receptors control the GABAergic synaptic activity in the DR, suggesting a localization of these receptors in presynaptic GABA terminals. Additionally, the deficient 5‐HT_1A_ and GABA_B_ receptor‐mediated presynaptic inhibition that GIRK2 KO mice exhibit points to a presynaptic modulation of GABA release by GIRK2 subunit‐containing channels. Although to the best of our knowledge there are no previous studies addressing directly the functional role of 5‐HT_1A_ receptors in the GABAergic synaptic activity, earlier electrophysiological reports demonstrated 5‐HT_1A_ receptor‐mediated responses in non 5‐HT neurons of the DR (Kirby et al. 2003; Beck et al. 2004). Likewise, anatomical studies detected 5‐HT_1A_ mRNA in GABAergic neurons (Day et al. 2004) and 5‐HT_1A_ receptor expression in 5‐HT‐immunonegative cells in the DR (Kirby et al. 2003). Given that 5‐HT_1B_ receptors have been demonstrated to control the GABA synaptic activity in the median, but not dorsal raphe (Lemos et al. 2006), our results may indicate that, besides 5‐HT_1B_ receptors and GABA_B_ receptors, 5‐HT_1A_ receptors modulate the GABAergic synaptic activity in the raphe nuclei. Whether GIRK channels play a presynaptic role in neurotransmitter release undoubtedly remains a controversial question. It is thought that presynaptic inhibition of neurotransmitter release results primarily, although not necessarily entirely, due to G protein‐dependent suppression of voltage gated Ca^2+^ channel activity (Dolphin 2003) or due to regulation of release downstream from Ca^2+^ influx (Scanziani et al. 1992; Sakaba and Neher 2003). Biochemical and morphological studies, however, have described presynaptic labeling of GIRK subunits, including GIRK2, in different brain structures (Ponce et al. 1996; Koyrakh et al. 2005; Marker et al. 2005; Ladera et al. 2008), and these findings were further supported by functional studies showing that GIRK channels control, at least in part, GABA release in the VTA and the cortex (Ladera et al. 2008; Michaeli and Yaka 2010). However, at least two studies failed to detect any presynaptic involvement of GIRK channels in neurotransmitter release in the hippocampus and periaqueductal gray (Luscher et al. 1997; Liu et al. 2012). We believe that the highly heterogeneous nature of GIRK channels regarding subunit composition depending on brain region, cell type and, likely, sub‐cellular localization (for review see Luscher and Slesinger 2010) accounts for the differing results obtained in the abovementioned studies. Our data suggest that, in the DR, GIRK channels regulate presynaptic and postsynaptic processes playing an important role in presynaptic neurotransmitter release and in the control of postsynaptic GPCR‐induced currents.

Two main findings in this study indicate that *Girk2* gene deletion not only affects presynaptic GABA_A_ receptor‐mediated signaling but also impedes postsynaptic GABA_A_ receptor activity. GIRK2 KO mice showed, first, reduced sIPSC amplitudes and, second, markedly reduced GABA‐induced fast inward currents compared to WT mice. These observations indicate that inactivation of the *Girk2* gene decreases the number, sensitivity, or time open of the activated GABA_A_ receptor (Kirby et al. 2008), likely because the well‐established regulation that postsynaptic GABA_B_ receptors exert over GABA_A_ receptor‐mediated currents is absent in the GIRK2 KO mice (Connelly et al. 2013; Tao et al. 2013). Consistent with our observations, two studies revealed that genetic inactivation of 5‐HT_1A_ and CB_1_ receptors_,_ which are coupled to GIRK channels, reduces the expression of certain GABA_A_ receptor subunits as well as the whole receptor functionality (Sibille et al. 2000; Uriguen et al. 2011). Having observed similar findings in the current work, it is tempting to speculate that the reduced GABA_A_ receptor expression and/or activity is a consequence of the lack of GIRK activity. Nonetheless, further studies are clearly required to unravel the interplay between GIRK2 and GABA_A_ receptor‐mediated transmission in the DR microcircuit.

Extending these results beyond physiological findings, growing evidence has identified GABAergic deficiencies in the prefrontal regions of unmedicated depressed patients and in animal models of depression induced by chronic social defeat stress (Hasler et al. 2007; Venzala et al. 2013). Importantly, Challis et al. (2013) showed that social defeat stress, which results in long‐lasting social avoidance and which is sensitive to the effects of antidepressants (Berton et al. 2006; Tsankova et al. 2006), suppresses 5‐HT neuronal firing through increased activity of GABA interneurons in the DR. Furthermore, decreasing GABA activity disinhibited 5‐HT cells and promoted a “resilient” phenotype in mice exposed to social defeat (Challis et al. 2013). Interestingly, these findings coincide with the reduced GABAergic activity observed here and with the previously reported increased 5‐HT neuronal firing and depression‐resistant phenotype that GIRK2 KO mice exhibit (Llamosas et al. 2015). Moreover, from our previous study and the present work, we revealed that mice lacking GIRK2 subunits show desensitized 5‐HT_1A_ receptors and reduced sIPSC frequency. Similarly, previous studies have reported that chronic treatment with fluoxetine, which desensitizes 5‐HT_1A_, and imipramine reduce GABAergic transmission in various regions of the cerebral cortex (Zhong and Yan 2004; Wabno and Hess 2013). In this regard, it is interesting that the alterations we detected in GIRK2 KO mice are opposite to those observed in socially defeated mice and mimic the effects of repeated antidepressant administrations on 5‐HT and GABAergic transmissions. Therefore, approaches targeting GIRK2 subunit in the DR may be a beneficial manipulation to treat or ameliorate stress‐related psychopathologies, such as anxiety and depression. This study presents a clear effect of GIRK channels on the GABAergic synaptic activity, but it is limited to electrophysiological observations in putative 5‐HT neurons. In the future, in order to strengthen the present conclusions, it could be of great value to inspect the pre‐ or postsynaptic localization of GIRK2 subunit‐containing channels in the different cells expressed in the DR and to study the contribution of GIRK channels to the physiology of this nucleus according to their cell type‐specific expression and function.

In conclusion, GIRK2 subunit‐containing channels play a primary role in controlling the electrophysiological properties and the 5‐HT and GABAergic systems in the DR. Given the high involvement of these neurotransmission systems of the DR in the pathophysiology of affective disorders, GIRK2 subunit‐containing channels may be valuable targets for studying the mechanisms underlying mood‐related disorders and for developing novel therapeutic agents.

## Conflict of Interests

The authors declare no conflict of interests.
